# The effect of 8-day oral taurine supplementation on thermoregulation during low-intensity exercise at fixed heat production in hot conditions of incremental humidity

**DOI:** 10.1007/s00421-024-05478-3

**Published:** 2024-04-06

**Authors:** Jennifer S. Peel, Melitta A. McNarry, Shane M. Heffernan, Venturino R. Nevola, Liam P. Kilduff, Kathryn Coates, Ed Dudley, Mark Waldron

**Affiliations:** 1 A-STEM Centre, Faculty of Science and Engineering, Swansea University, Swansea, UK; 2 Defence Science and Technology Laboratory (Dstl), Fareham, Hampshire UK; 3 Welsh Institute of Performance Science, Swansea University, Swansea, UK; 4 Swansea University Medical School, Faculty of Medicine, Health and Life Science, Swansea University, Swansea, UK; 5 School of Health and Behavioural Sciences, University of the Sunshine Coast, Sippy Downs, QLD Australia

**Keywords:** Taurine, Sweating, Heat, Uncompensability, Evaporative cooling, Thermoregulation

## Abstract

**Purpose:**

To determine the effect of taurine supplementation on sweating and core temperature responses, including the transition from compensable to uncompensable heat stress, during prolonged low-intensity exercise of a fixed-heat production (~ 200W/m^2^) in hot conditions (37.5 °C), at both fixed and incremental vapour-pressure.

**Methods:**

Fifteen females (*n* = 3) and males (*n* = 12; 27 ± 5 years, 78 ± 9 kg, $\dot{V}$O_2max_ 50.3 ± 7.8 mL/kg/min), completed a treadmill walking protocol (~ 200W/m^2^ heat production [*Ḣ*_prod_]) in the heat (37.5 ± 0.1 °C) at fixed-(16-mmHg) and ramped-humidity (∆1.5-mmHg/5-min) following 1 week of oral taurine supplementation (50 mg/kg/bm) or placebo, in a double-blind, randomised, cross-over design. Participants were assessed for whole-body sweat loss (WBSL), local sweat rate (LSR), sweat gland activation (SGA), core temperature (*T*_core_), breakpoint of compensability (*P*_crit_) and calorimetric heat transfer components. Plasma volume and plasma taurine concentrations were established through pre- and post-trial blood samples.

**Results:**

Taurine supplementation increased WBSL by 26.6% and 5.1% (*p* = 0.035), LSR by 15.5% and 7.8% (*p* = 0.013), SGA (1 × 1 cm) by 32.2% and 29.9% (*p* < 0.001) and SGA (3 × 3 cm) by 22.1% and 17.1% (*p* = 0.015) during the fixed- and ramped-humidity exercise periods, respectively. Evaporative heat loss was enhanced by 27% (*p* = 0.010), heat-storage reduced by 72% (*p* = 0.024) and *P*_crit_ was greater in taurine *vs* placebo (25.0-mmHg vs 21.7-mmHg;* p* = 0.002).

**Conclusion:**

Taurine supplementation increased sweating responses during fixed *Ḣ*_prod_ in hot conditions, prior to substantial heat strain and before the breakpoint of compensability, demonstrating improved thermoregulatory capacity. The enhanced evaporative cooling and reduced heat-storage delayed the subsequent upward inflection in *T*_core_—represented by a greater *P*_crit_—and offers a potential dietary supplementation strategy to support thermoregulation.

**Supplementary Information:**

The online version contains supplementary material available at 10.1007/s00421-024-05478-3.

## Introduction

Exercise increases metabolic heat production (*Ḣ*_prod_), with dry (conduction, convection and radiation) and evaporative heat exchange providing potential avenues of heat dissipation (Gagge and Gonzalez 1996). In hot environmental conditions, evaporative heat transfer (latent heat transfer; *Ė*_skin_) is the main and most modifiable avenue of heat dissipation, which occurs secondary to sweating when ambient vapor pressure permits (Wenger 1972). Indeed, both *Ḣ*_prod_ and the rate of evaporation required to maintain heat balance (*Ė*_req_) drive the thermal sweating response (Cramer and Jay 2014, 2016; Gagnon et al. 2013; Peel et al. 2022). Consequently, when exercising or performing occupational work in the heat, eccrine sweat production is an important physiological mechanism for the maintenance of thermal equilibrium, as it allows for evaporative cooling to offset *Ḣ*_prod_ (Marino et al. 2000; Sawka and Young 2006). Thus, manipulation of factors affecting calorimetric components, such as *Ḣ*_prod_ or any heat loss avenue, will affect thermal balance.

Evaporation of sweat off the skin’s surface, and therefore, the latent heat of vaporisation, is determined by both the efficiency of sweating and the ambient vapour pressure [determined from the relationship between air temperature and relative humidity (RH); Gagge and Gonzalez 1996; Parsons 2007]. Dry, hot environmental conditions allow for a greater capacity to evaporatively cool compared to those with high humidity (Che Muhamed et al. 2016). This is due to the larger vapour pressure gradient between the ambient air and skin’s surface, which permits a superior maximal evaporative heat transfer capacity (*Ė*_max_; Cramer and Jay 2019). Consequently, prolonged exercise in hot and humid environmental conditions causes thermal strain (positive heat storage), due to the reduced capacity for evaporative cooling. Indeed, without sufficient dry and evaporative heat transfer, positive heat storage will ensue, forcing a transition from a compensable to an uncompensable state, denoted by inexorable increases in core temperature (*T*_core_; Cramer and Jay 2016; Marino et al. 2000). Thermal sweating and the efficiency of evaporative heat transfer can be acutely enhanced through interventions, such as endurance training and heat acclimation (Ravanelli et al. 2018). This training- or acclimation-induced augmentation of the sweating response was reported to delay the upward inflection in *T*_core_ associated with a transition to thermal uncompensability using an ‘inflection protocol’, whereby ambient vapor pressure is manipulated at fixed ambient dry bulb temperature and heat production (Ravanelli et al. 2018). Thus, manipulation of the environmental conditions can be useful in determining capacity to thermoregulate and changes that might occur following interventions, such as dietary supplementation.

Taurine, a sulphur containing amino acid, can be supplemented orally and has been shown to enhance endurance exercise performance in thermoneutral conditions (Balshaw et al. 2013; Waldron et al. 2019, 2018b; Zhang et al. 2004b). These ergogenic effects mimic the magnitude of endurance training responses to heat acclimation (Waldron et al. 2021) and appear to be related to sarcoplasmic reticulum Ca^+^ handling (Dutka et al. 2014; Hamilton et al. 2006), anti-oxidative effects (Hansen et al. 2006, 2010; Jong et al. 2021; Schaffer et al. 2022; Zhang et al. 2004b) and/or alterations in substrate utilisation, favouring greater relative fat oxidation (Rutherford et al. 2010; Simmonds et al. 2022), which is a feature of the endurance trained phenotype (Lima-Silva et al. 2010). Further, taurine has many other biological roles that could be advantageous to exercise performance in the heat, including cellular osmoregulation (Cuisinier et al. 2002) and vasoactive properties (Maia et al. 2014; Sun et al. 2016; Ulusoy et al. 2017). The osmoregulatory capacity of taurine might be exaggerated following oral supplementation, with higher plasma taurine concentrations increasing osmotic pressure in both central and peripheral sites, thereby acutely drawing fluid into the vascular space and theoretically sustaining, or perhaps expanding, plasma volume. However, it should be noted that taurine has a weaker relationship with plasma volume compared to other osmolytes, such as sodium and chloride (Cuisinier et al. 2002) and the effect of exogenous supplementation on plasma volume in heat stressed, exercising humans has not yet been established. Nonetheless, various beneficial physiological effects of taurine supplementation have been demonstrated in hot conditions, as described below. Indeed, the performance enhancing effect of taurine supplementation was heightened when administered during exercise in the heat, prolonging time to exhaustion by 10% (Page et al. 2019). Here, taurine increased the rate (∼12.7%) and hastened the onset of sweating during exhaustive exercise in the heat, alongside substantial reductions in *T*_core_ (end *T*_core_ of 38.1 °C vs 38.5 °C), demonstrating its potential role in thermoregulation. The early changes in the sweating response were considered to be indicative of a centrally mediated alteration in thermoregulatory set-points, which might relate to its role as a neuromodulator (Hussy et al. 2000; Jia et al. 2008). However, despite this early promising research, it remains necessary to more comprehensively evaluate the potential thermoregulatory role of taurine supplementation in a controlled experimental setting, where sufficient control of calorimetric components is permitted (i.e. *Ḣ*_prod_ and *Ė*_req_). Furthermore, the effect of taurine on the sweating response and subsequent thermoregulation across more prolonged exercise periods is unknown.

The aim of the current study was to determine the effect of an 8-day taurine supplementation period on *T*_core_ and sweating responses [whole-body sweat loss (WBSL), local sweat rate (LSR) and sweat gland activation (SGA)], calorimetric heat transfer components (*Ė*_skin_, heat storage), delta plasma volume, and plasma taurine concentrations during prolonged low-intensity exercise of a fixed *Ḣ*_prod_ in the heat at both fixed and increasing vapour pressure. It was hypothesised that taurine supplementation would: (i) induce greater sweating responses across the exercise protocol; (ii) delay the increase in *T*_core_ during the period of increasing vapour pressure (transition to an uncompensable environment); (iii) increase plasma volume and; (iv) result in greater evaporative heat transfer and reduced heat storage, as modelled by partitional calorimetry.

## Methods

### Participants

Fifteen non-heat acclimated, healthy females (*n* = 3) and males (*n* = 12) volunteered to take part in the study [27 ± 5 years, 179 ± 8 cm, 78 ± 9 kg, maximal oxygen uptake ($\dot{V}$O_2max_) 50.3 ± 7.8 mL/kg/min]. Based on the effect sizes (Cohen’s *d* = 0.7) reported using taurine to improve endurance performance in the heat (Page et al. 2019), G ∗ Power (Version 3.0.10; Universität Düsseldorf, Germany) was used to calculate an appropriate *a-priori* sample size of 15 to identify significant differences between groups. As part of the health screening questionnaire, participants were asked if they had been exposed to hot ambient temperatures in the previous two months, sufficient to induce heat adaptation and were excluded if so. Participants were asked to refrain from alcohol and caffeine consumption for 24-h and to avoid strenuous exercise and follow a consistent diet for 48-h prior to testing. Use of any performance enhancing or dietary supplements, such as caffeine, was prohibited for the duration of the study. This was verified in the pre-trial screen, along with the opportunity for participants to report any adverse health effects. Written informed consent was obtained from all participants. Institutional ethics approval (JP_30-10-20b) was provided and the study was conducted in accordance with the 2013 Declaration of Helsinki, except for pre-registration on a publicly accessible database.

### Design

**Fig. 1:**
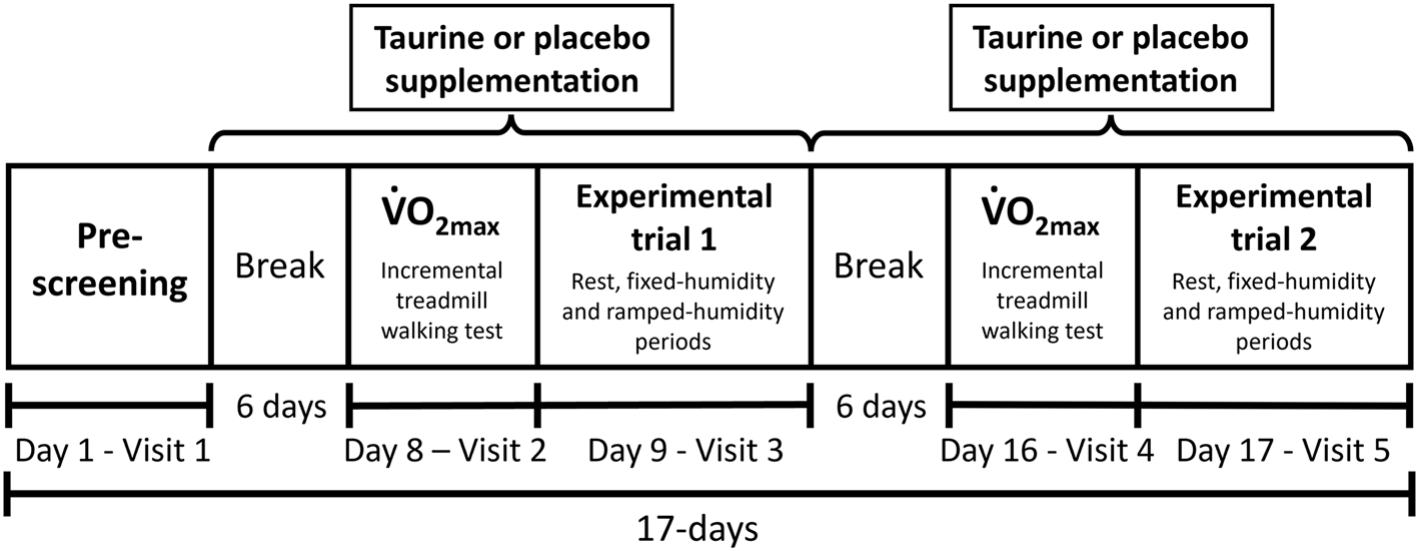
Schematic of the study timeline. $\dot{V}$O_2max =_ maximal oxygen uptake

This study adopted a double-blind, randomised, placebo-controlled, cross-over design. Participants reported to the laboratory on five separate occasions; once for pre-screening and familiarisation (visit 1), twice to complete a walking incremental test to establish the work rate-$\dot{V}$O_2_ relationship and $\dot{V}$O_2max_ (visits 2 and 4) and twice for the experimental trials, in which they completed a fixed- and ramped-humidity treadmill walking protocol, following 8-days of supplementation with either 50 mg/kg/bm of taurine or 30 mg/kg/bm of maltodextrin (placebo; visits 3 & 5; Fig. 1). All testing sessions took place in an environmental chamber set to 37.5 ± 0.1 °C and 34.2 ± 1.4% RH. The break period of 7-days between conditions was selected to permit complete recovery from the protocols and time to consume the cross-over supplementation. Taurine has a ratio of clearance/bioavailability of ~ 21-h and, therefore, this was considered a sufficient washout period (Ghandforoush-Sattari et al. 2010). All trials were conducted at approximately the same time of day to control for circadian rhythm variation. Randomisation was performed manually via coin toss by an independent person.

### Incremental walking test

During visits 2 and 4, participants completed an incremental treadmill walking test to volitional exhaustion on a calibrated treadmill (h/p/cosmos, Am Sportplatz 8, Germany) in hot conditions (37.5 ± 0.1 °C and 34.2 ± 1.5% RH) to determine their $\dot{V}$O_2max_ and individual work rates required to elicit *Ḣ*_prod_ of ~ 200 W/m^2^. The test began at 2 km/h (0.56 m/s) and increased by 1 km/h (0.28 m/s) every 3-min with corresponding gradients of 0% in the first stage, 5% in the second stage, 10% in the third stage, and 2% additional increases thereafter. The incremental test was conducted to volitional exhaustion. Pulmonary oxygen uptake was measured using breath-by-breath expired gas analysis (Jaeger Vyntus CPX, Hoechberg, Germany), with $\dot{V}$O_2max_ determined as the highest 30-s mean value, which occurred in the final stage of each participants’ test. The test was designed to progressively increase mechanical work rate, in a square-wave manner, to elicit a range of *Ḣ*_prod_ values, including that required for the treadmill walking protocol in visits 3 and 5 (200 W/m^2^). Each participant’s *Ḣ*_prod_ for the experimental trials were determined by subtracting the rate of mechanical work (Wk) from the rate of metabolic energy expenditure (*Ṁ*; Eq. 1).

1 $${\dot{H}}_{{\text{prod}}}={\dot{M}}-{\dot{W}}{\text{k}} \,\, [{\text{W}}]$$

where: metabolic energy expenditure (*Ṁ*) was determined using measured $\dot{V}$O_2_ and respiratory exchange ratio (RER) in the final 1-min of each incremental stage (Eq. 2):

2 $${\dot{M}}= {\dot{{\text{V}}}{\text{O}}}_{2}\times \frac{\left(\left(\frac{{\text{RER}}-0.7}{0.3}\right)\times 21.13\right)+ \left(\left(\frac{1.0-{\text{RER}}}{0.3}\right)\times 19.62\right)}{60}\times 1000\, [{\text{W}}]$$

To achieve the necessary *Ḣ*_prod_ value, the test was initiated at a mechanical work rate below that required to elicit the desired *Ḣ*_prod_ (200 W/m^2^) and increased until exhaustion. The *Ḣ*_prod_ (W/m^2^) at each stage was determined based on participant body surface area (BSA; Eqs. 3 and 4; (Cramer and Jay 2014; DuBois and DuBois 1916).

3 $${{\dot{H}}}_{{\text{prod}}}=\frac{{{\dot{H}}}_{{\text{prod}}}}{{\text{BSA}}} [{\text{W}}/{{\text{m}}}^{2}]$$

4 $${\text{BSA}}=0.00718\times ({\mathrm{body\, mass\, }\left({\text{kg}}\right)}^{0.425})\times \left({\mathrm{height\, }({\text{cm}})}^{0.725}\right) [{{\text{m}}}^{2}]$$

The mechanical work rate required to elicit each target *Ḣ*_prod_ (W/m^2^) for the exercise trials during visits 3 and 5 (i.e. ~ 200 W/m^2^) was subsequently determined based on the linear relationship (*y* = *mx* + *b*), between *Ḣ*_prod_ (W/m^2^) and work rate during the incremental test (Eq. 5). This equated to ~ 43.1 ± 6.2 W and ~ 34.7 ± 5.5% of $\dot{V}$O_2max_ in the taurine condition and ~ 42.1 ± 6.2 W and ~ 34.9 ± 5.6% of $\dot{V}$O_2max_ in the placebo condition.

5 $$\mathrm{Required \,work\, rate}=\frac{\mathrm{Desired\, }{{\dot{H}}}_{{\text{prod}}} ({\text{W}}/{{\text{m}}}^{2}) - y\mathrm{\, intercept}}{{\text{Slope}}}\, [{\text{W}}]$$

### Experimental trials

#### Pre-trial instrumentation

Participants were required to arrive euhydrated, as determined by a urine osmolality value < 600 mOsm kg/H_2_O (Portable osmometer, Osmocheck, Vitech, Scientific Ltd). If the reading was > 600 mOsm kg/H_2_O—the threshold for hypohydration—the participant was asked to drink 500-mL of plain water and wait 30-min. Urine osmolality was then re-determined and if the participant was deemed euhydrated, testing commenced. Participants wore running shorts (90% polyester, 10% elastane), as well as a sports bra for female participants. To measure core body temperature (*T*_core_), participants were instructed to insert a flexible rectal probe 10 cm past the anal sphincter (Walters Medical, W0001B, England).

#### Trials (rest, fixed-humidity and ramped-humidity)

Participants initially rested for 30-min in a seated position within the environmental chamber, which was regulated to an ambient dry bulb temperature (*T*_db_) of 37.5 ± 0.1 °C, RH of 34.2 ± 1.4% and vapour pressure of 16-mmHg. Environmental conditions, such as ambient *T*_db_, RH and air velocity (m/s), were continuously monitored approximately 120 cm from the exercising participant (Kestrel 5400 Heat Stress Tracker, Kestrel Meters, Boothwyn, PA, US). A large electric fan (SIP 24” Drum Fan, Loughborough, UK) was placed in front of the participant during the rest and exercise periods, providing an airflow of 2.0 ± 0.2 m/s, directed at the torso. During the rest period, skin thermistors (Grant Instruments Ltd., Cambridge, UK) were attached to four sites on the participant’s left side: upper chest, mid-humerus, mid-calf and mid-thigh to measure weighted mean skin temperature (*T*_sk_). Prior to application of the skin thermistors, the skin was dry-shaved. Both *T*_core_ and *T*_sk_ temperature were recorded using a data logger, continuously sampling every 5-s (SQ2010; Grant Instruments Ltd., Cambridge, UK). Ramanathan’s equation (Ramanathan 1964) was used to calculate mean *T*_sk_:

6 $${T}_{{\text{sk}}}= \left({T}_{{\text{chest}}}+{T}_{{\text{arm}}}\right)\times 0.3+\left({T}_{{\text{thigh}}}+{T}_{{\text{calf}}}\right)\times 0.2 [^\circ {\text{C}}]$$

**Fig. 2:**
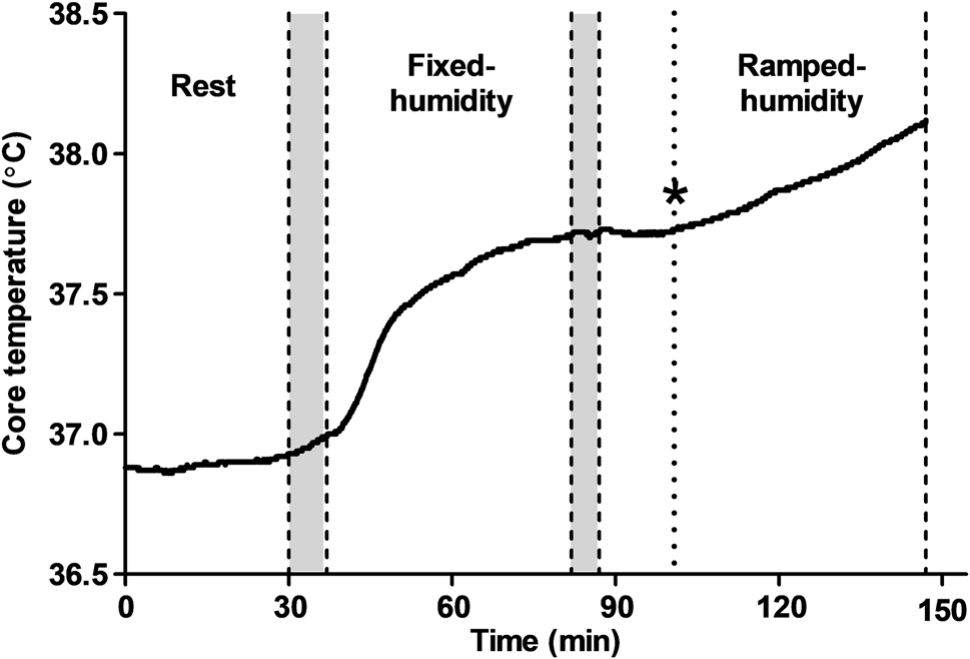
Example core temperature time series of a representative participant during the experimental trial. Asterisk: point of inflection, grey bars = transition periods

**Fig. 3:**
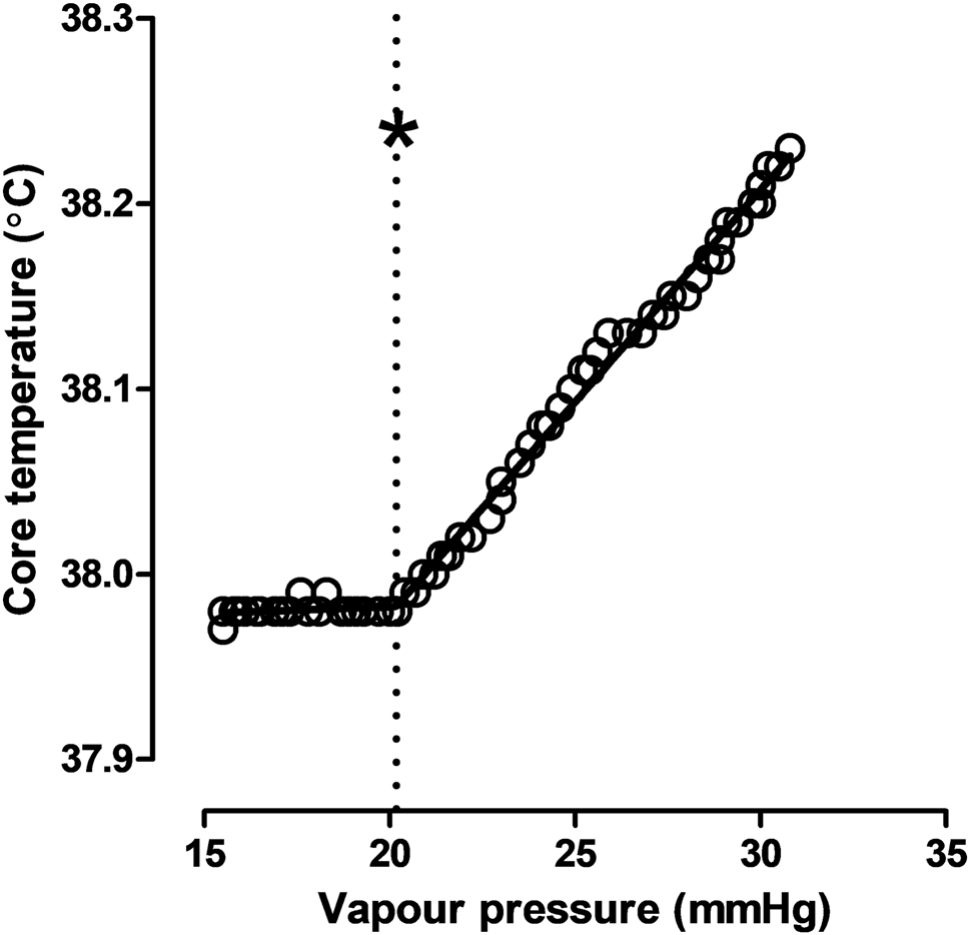
The ambient vapour pressure at the point of upward inflection in core temperature. Asterisk: point of inflection

After 30-min of rest in the chamber, the participants began walking on the treadmill at an individual-specific speed and gradient intended to elicit a pre-determined fixed *Ḣ*_prod_ (~ 200 W/m^2^). After 45-min of exercise (fixed-humidity exercise period), the ambient vapour pressure (mmHg) inside the environmental chamber increased by 1.5-mmHg every 5-min for an additional 60-min (ramped-humidity exercise period). The point at which an upward inflection in *T*_core_ was observed was identified as the critical ambient vapour pressure (*P*_crit_), theoretically indicating the transition from a compensable to an uncompensable state (Kenney and Zeman 2002; Ravanelli et al. 2018); Figs. 2 and 3). The inflection in *T*_core_ at the breakpoint of compensability (*P*_crit_) was determined using segmental linear regression of the *T*_core_—ambient vapour pressure relationship, which was averaged to 1-min values during the ramped-humidity exercise period (Graphpad Prism, version 5.01, La Jolla, CA). Participants were provided with 200-mL of plain water [maintained at room temperature (~ 20 °C)] after the rest period and before exercise, and 400-mL between the 45-min fixed-humidity and 60-min ramped-humidity exercise periods. Fluid intake was later accounted for when determining changes in body mass losses at selected trial stages.

During exercise, $\dot{V}$O_2_ was measured using the same breath-by-breath gas analyser. Heart rate (HR) was recorded throughout (Polar Heart Rate Monitor M400, Warwick, UK). Rating of perceived exertion (RPE) was recorded using a 6–20-point Borg scale (Borg 1982), while thermal comfort (TC) was recorded using a 7-point scale [where −3 = “much too cool”, 0 = “comfortable” and 3 = “much too warm” (Bedford 1936)]. Thermal sensation (TS) was recorded using a 9-point scale [where −4 = “very cold”, 0 = “neutral” and 4 = “very hot” (Zhang et al. 2004a)]. Perceptual data (RPE, TC and TS) were recorded at 5-min intervals during the rest and exercise periods.

#### Sweating measurements

Participants’ body mass was measured at multiple timepoints during the trial. Given the nature of the exercise trials, participants’ body mass was recorded whilst wearing cycling shorts, a sports bra for women, a HR monitor, with the inserted rectal thermistor and the skin thermistors fitted to the skin. Whilst this added some mass to the participant, this was consistent throughout all trials. A force plate (Type 1758A10, Kistler Instruments Ltd, Farnborough, UK) was used at sampling frequency of 1000-Hz and had a coefficient of variation of 0.05% for body mass measurements. Participants’ body mass was measured in the environmental chamber, on a hard, flat surface, immediately pre-exercise and post the 45-min fixed-humidity and 60-min ramped-humidity exercise periods.

Local sweat rate was determined using the absorbent patch technique on the left scapula, via the method reported by Peel et al. (2022). Measurements were taken during the final 5-min of the 45-min fixed-humidity exercise period and the 60-min ramped-humidity exercise period. The patch (Medipore + Pad [3 M]) was 5 cm × 5.5 cm, with an absorbent capacity of ~ 7-g. It was weighed (resolution 0.01-g; Ohaus, Navigator N24120, Nänikon Switzerland) prior to and after the 5-min skin application. Local sweat rate (mg/cm^2^/min) was determined using Eq. (7).

7 $$\mathrm{Local \,sweat \,rate}= \frac{\mathrm{pre \,to \,post \,change \,in \,patch \,mass }\,({\text{mg}})}{[5 \,\left({\text{cm}}\right) \times 5.5\, \left({\text{cm}}\right)] \times 5 ({\text{min}})} [{\text{mg}}/{{\text{cm}}}^{2}/{\text{min}}]$$

The modified iodine-paper technique was used to determine SGA on the right scapula, using the method detailed in Peel et al. (2022). In brief, 100% cotton paper (Southworth, Agawam, MA, US) was cut to 9 × 9 cm, saturated with iodine in the preceding 24-h, and then applied to the skin for 5-s at the end of the 45-min fixed-humidity exercise period and the 60-min ramped-humidity exercise period. As recommended by Peel et al. (2022), to establish SGA, the optimal area of sweat gland density within 3 × 3 cm and 1 × 1 cm areas within the 9 × 9 cm iodine paper area was determined. The optimal area was defined as the area (3 × 3 cm and 1 × 1 cm) with the highest density of recruited glands.

#### Partitional calorimetry

As detailed in supplementary material (Online Resource 1), heat balance parameters, such as *Ḣ*_prod_, evaporative requirement for heat balance (*Ė*_req_), evaporation at the skin surface (*Ė*_skin_) and heat storage (*S*; Eqs. 7–10) were estimated via partitional calorimetry (Cramer and Jay 2019). *Ḣ*_prod_ was also expressed relative to body surface area (DuBois and DuBois 1916).

8 $${{{\dot{E}}}}_{{\text{req}}}= {{\dot{H}}}_{{\text{prod}}}- {{\dot{H}}}_{\mathrm{dry skin}}- {{\dot{H}}}_{{\text{res}}} [{\text{W}}]$$

9 $${{\dot{E}}}_{{\text{skin}}}=\mathrm{ delta\, body \,mass \,loss}\times \frac{\lambda }{1000} [{\text{kJ}}]$$

where: *λ* is the latent heat vaporisation of sweat (2426 J/g).

10 $${\text{S}}=\mathrm{ time}\times \frac{{{\dot{H}}}_{{\text{prod}}} - {{\dot{H}}}_{\mathrm{dry skin}} - {{\dot{H}}}_{\mathrm{evap skin}} - {{\dot{H}}}_{{\text{res}}}}{1000} [{\text{kJ}}]$$

On the assumption that blood entering and leaving the cutaneous circulation was equal to core and skin temperatures, respectively, maximum skin blood flow (SkBF) was determined as (Sawka and Young 2006):

11 $${\text{SkBF}}=\frac{(\frac{1}{{\text{SH}}}\times {{\dot{H}}}_{{\text{prod}}})}{{(T}_{{\text{core}}}-{T}_{{\text{sk}}})} [{\text{ND}}]$$

where SH is the specific heat of the blood (~ 1 kcal/°C) and *Ḣ*_prod_ is expressed in kcal/min.

### Supplementation

After randomisation to the placebo or experimental condition, all supplements were administered in powder form within gelatine capsules, in a double-blind manner. The capsules contained either 100% isolated taurine or placebo (100% maltodextrin) and were prepared using an analytical balance (Ohaus, Navigator N24120, Nänikon Switzerland). Participants ingested the supplements for a total of 8-days, having 50 mg/kg of body mass per day of taurine or 30 mg/kg of body mass per day of maltodextrin across the 8-day period. On day 7 of supplementation, participants performed the incremental test and ingested the supplements 1.5-h prior to exercise. On day 8 of supplementation, the participants undertook the experimental trial and ingested the supplements 30-min before entering the environmental chamber. Supplement blinding was deemed successful, as participants only guessed which condition they were in correctly 33% of the time. The taurine dosage administered in the current study was informed by published recommendations (Waldron et al. 2018b; Warnock et al. 2017) and because it has previously been demonstrated to be efficacious for thermal sweating during exercise in the heat (Page et al. 2019). The timing of ingestion was designed to elicit peak plasma taurine availability during exercise in both the incremental test and the experimental trial (Ghandforoush-Sattari et al. 2010). Both the taurine and maltodextrin were sourced from Myprotein (Manchester, UK).

#### Blood sampling

Venous blood samples were taken pre- and post-trial for the measurement of plasma taurine concentration and fingertip capillary blood samples were used to estimate plasma volume changes (Dill and Costill 1974). Both pre- and post-measurements were conducted in a cool room (~ 20 °C). Participants were asked to sit quietly for 10-min prior to any blood sampling, as plasma volume is affected by postural changes (Hagan et al. 1978). Blood was drawn into capillary tubes and microcuvettes (Hemocue Hb 201) for the measurement of haematocrit and haemoglobin concentration, respectively. The capillary tubes were spun in a microcentrifuge (Hawksley Neuation HCT Hematocrit Centrifuge, iFuge-HCT, Hawksley & Sons Ltd., Sussex, England) at 10,000 rev/min for 5-min and separated red cell volume was measured using a haematocrit reader (Hawksley Micro-Haematocrit Reader, Hawksley & Sons Ltd., Sussex, England). All samples were taken and measured in duplicate, with the mean value recorded for analysis. Venous blood samples were obtained via venipuncture from an antecubital vein and were drawn into three EDTA-treated vacutainer tubes (6-mL). These tubes were immediately placed on ice for 15-min before being centrifuged at 3000 rev/min for 15-min at 4 °C. The plasma was pipetted into 1.5-mL eppendorfs and stored in a − 80 °C freezer for subsequent analysis of taurine concentration.

#### Blood analysis

Plasma taurine concentration was measured using high performance liquid chromatography (HPLC). 100-µL of plasma sample was depleted through the addition of 400-µL of methanol and vortexed for 10-min before being centrifuged at 3000 rev/min for 5-min. The supernatant was speed vacuum concentrated to dryness at 7 °C and reconstituted in 100 µL of 0.4 M (pH 9) sodium bicarbonate buffer before being spiked with aspartic acid standard. The samples were analysed for taurine content using an agilent 1100 system utilising a pre-column derivatisation process and utilising OPA reagent. The samples were separated using a C18 column and ran using a gradient elution of 40-mM sodium phosphate buffer (pH 7.8) and ACN:MeOH:H_2_O (45:45:10) at 40 °C at a flow rate of 1 mL/min. Taurine that was successfully derivatised was detected using the fluorescence detector excitation 240-nm an emissions 450-nm with a PMT gain: 10 and a peak width of 0.5-min. Peak heights were used for quantifications.

### Statistical analysis

A two-way repeated measures analysis of variance (RM-ANOVA) was conducted with time (rest, fixed-humidity and ramped-humidity exercise periods) and condition (taurine and placebo) as the independent variables [WBSL, LSR, SGA, *T*_core_, *T*_sk_, TC, TS, RPE, HR, plasma (tau) concentration]. A Greenhouse–Geisser correction was applied when the assumption of sphericity was violated. Post hoc analysis was conducted with Bonferroni correction to identify significant pairwise comparisons if significant interaction effects were observed. Data were checked for normality using the Shapiro–Wilk test (Shapiro and Wilk 1965). Two-tailed paired samples *t* tests were used to identify significant differences between trials ($\dot{V}$O_2peak_, *P*_crit_, *T*_core_ at *P*_crit_, delta *T*_sk_ during ramped-humidity, *Ė*_skin_, and heat storage during fixed-humidity and plasma volume). A Wilcoxon Signed-Rank test was performed on non-parametric data [delta *T*_core_ during ramped-humidity, skin blood flow (SkBF)]. All statistical analysis was conducted in SPSS (IBM SPSS Statistics for Windows, IBM Corp, Version 24.0. Armonk, New York). Data are expressed as means ± SD throughout and a significance level of *p* < 0.05 was accepted across all tests. The magnitude of effects was calculated using Cohen’s *d* and partial eta squared (*η*_p_^2^) using the following criteria of 0.2 and 0.02 (small effect); 0.5 and 0.13 (medium effect); and 0.8 and 0.26 (large effect) to denote differences, respectively (Cohen 1988).

## Results

### Thermo-physiological responses

**Fig. 4:**
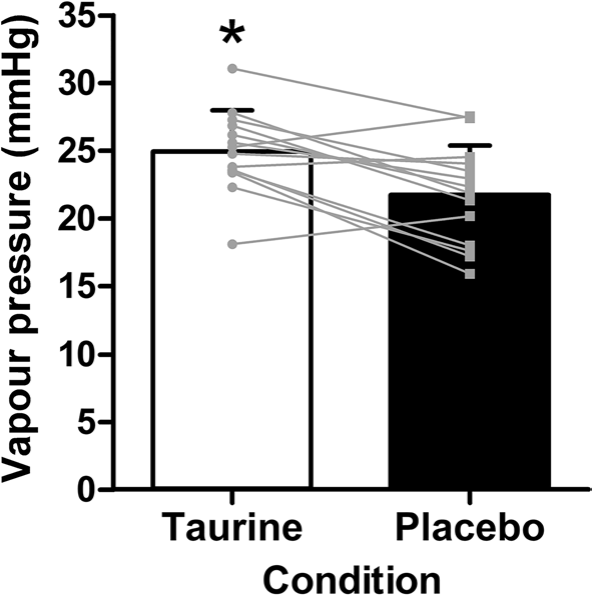
Critical ambient vapour pressure (*P*_crit_)—the point at which an upward inflection in core temperature was observed—indicating the transition from a compensable to an uncompensable state during the ramp-humidity exercise period in taurine and placebo conditions (mean ± SD). Asterisk: significantly greater than placebo (*p* < 0.05)

*P*_crit_ was greater in the taurine condition compared to placebo (*t*_(13)_ = 3.817, *p* = 0.002, Cohen’s *d* = 0.97; Fig. 4). However, there was no difference in *T*_core_ at the point of inflection (*t*_(13)_ = −0.046, *p* = 0.964, Cohen’s* d* = −0.01) or in delta *T*_core_ during the ramped-humidity exercise period (*p* = 0.624) between conditions.

**Fig. 5:**
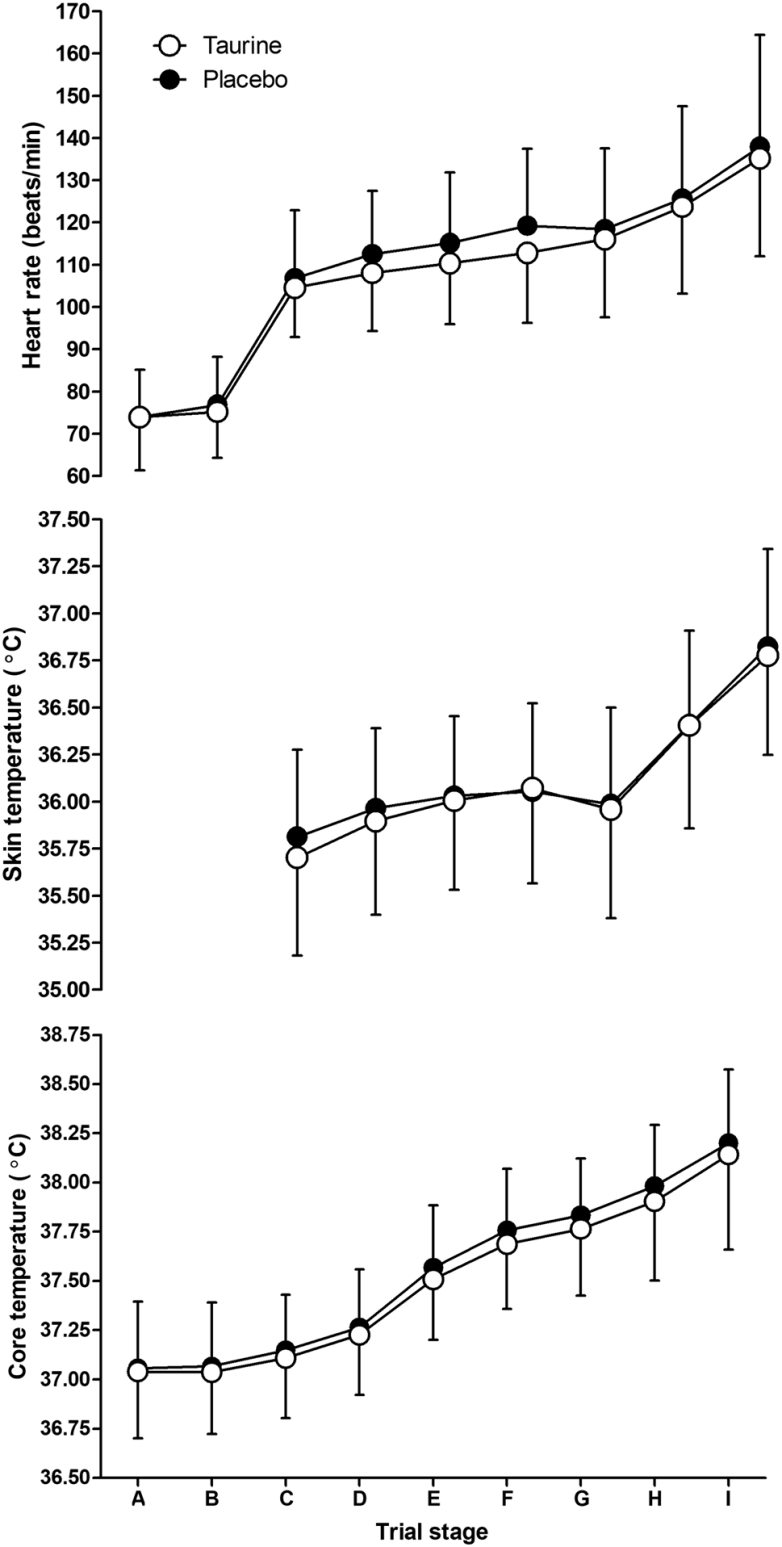
Heart rate (top), skin temperature (middle) and core temperature (bottom) plotted across time in the experimental trials (mean ± SD). **A** Start, **B** rest mean, **C** fixed-humidity start, **D** fixed-humidity stage 1 mean, **E** fixed-humidity stage 2 mean, **F** fixed-humidity stage 3 mean, **G** ramped-humidity start, **H** ramped-humidity mean, **I** end. **A**, **B** are intentionally missing for skin temperature

At rest and during the fixed-humidity exercise period, *T*_core_ increased with time across both conditions (*F*_(1.508, 21.117)_ = 236.585, *p* < 0.001,* η*_p_^2^ = 0.944); however, there was no main effect of condition (*F*_(1, 14)_ = 0.398, *p* = 0.538,* η*_p_^2^ = 0.028) or an interaction between condition and time (*F*_(1.394, 19.103)_ = 0.394, *p* = 0.601,* η*_p_^2^ = 0.027). During the fixed-humidity exercise period, mean *T*_sk_ increased with time in both conditions (*F*_(1.150, 16.106)_ = 15.779, *p* < 0.001,* η*_p_^2^ = 0.530); however, there was no main effect of condition (*F*_(1, 14)_ = 0.975, *p* = 0.340,* η*_p_^2^ = 0.065) or an interaction effect with time (*F*_(1.227, 17.173)_ = 3.668, *p* = 0.065,* η*_p_^2^ = 0.208). There was also no difference in the change in mean *T*_sk_ within the ramped-humidity exercise period (*t*_(14)_ = −0.435, *p* = 0.670, Cohen’s* d* = −0.08). Heart rate increased with time in both conditions during the whole trial (*F*_(1.281, 17.937)_ = 95.916, *p* < 0.001,* η*_p_^2^ = 0.873); however, there was no main effect of condition (*F*_(1, 14)_ = 2.134, *p* = 0.166,* η*_p_^2^ = 0.132) or an interaction effect (*F*_(2.354, 32.956)_ = 3.101, *p* = 0.051,* η*_p_^2^ = 0.181; Fig. 5).

There was no difference in $\dot{V}$O_2peak_ taken during the incremental walking test in visits 2 and 4 (Taurine = 50.6 ± 8.0 mL/kg/min; Placebo = 50.1 ± 7.8 mL/kg/min; *t*_(14)_ = 1.886, *p* = 0.080, Cohen’s* d* = 0.08) between conditions.

### Sweating measurements

**Fig. 6:**
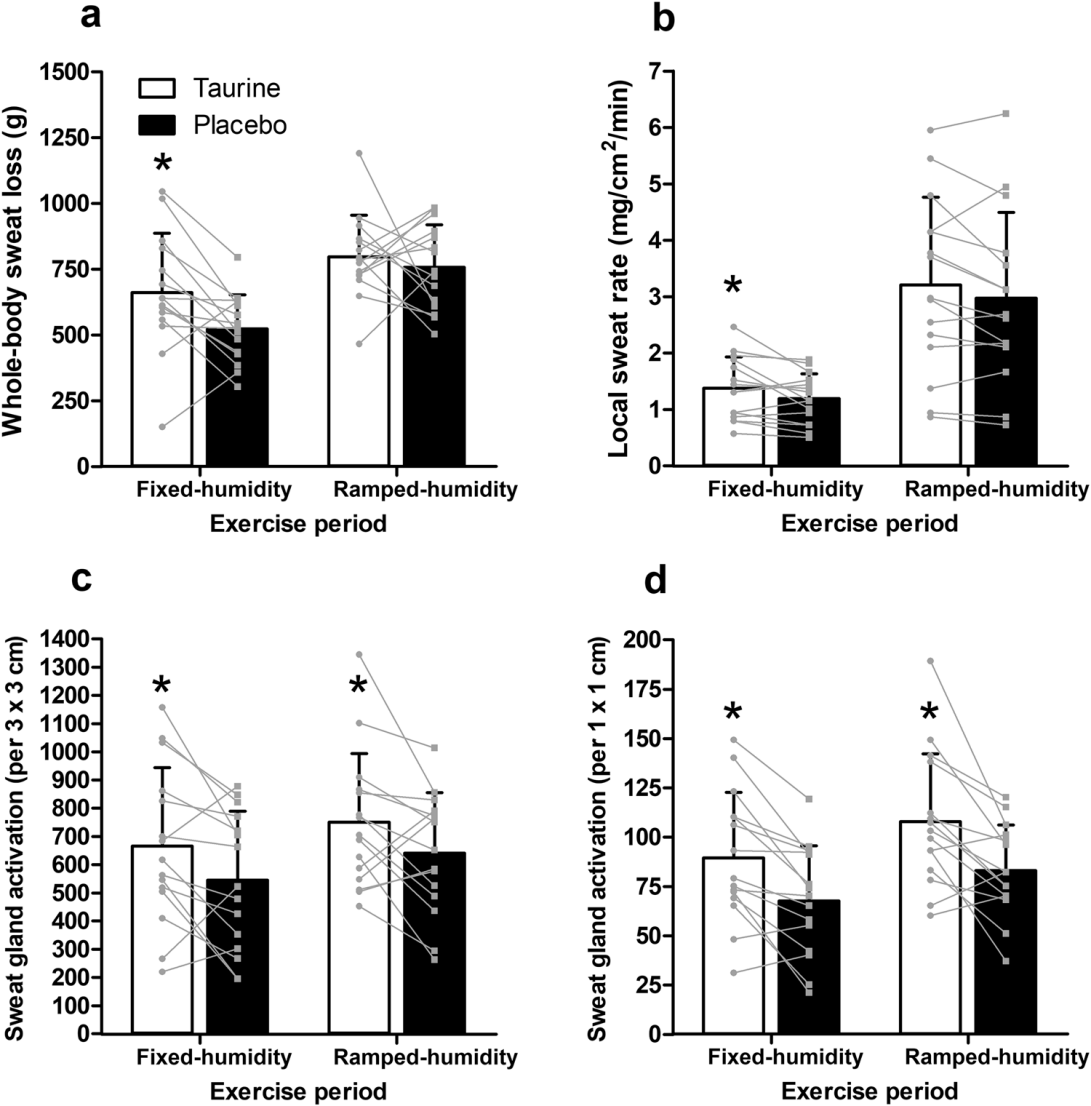
Whole-body sweat loss (**a**), local sweat rate (**b**), sweat gland activation (3 × 3 cm; (**c**) and (1 × 1 cm) (**d**) during fixed-humidity and ramped-humidity exercise periods (mean ± SD). Asterisk: significantly greater than placebo (*p* < 0.05)

All sweating variables are displayed in Fig. 6. Whole-body sweat loss (*F*_(1, 14)_ = 5.425, *p* = 0.035,* η*_p_^2^ = 0.279), local sweat rate (*F*_(1, 14)_ = 8.124, *p* = 0.013,* η*_p_^2^ = 0.367) and sweat gland activation (optimal 3 × 3 cm [*F*_(1, 14)_ = 7.750, *p* = 0.015,* η*_p_^2^ = 0.356] and 1 × 1 cm [*F*_(1, 14)_ = 22.525, *p* < 0.001,* η*_p_^2^ = 0.617]) were significantly increased in the taurine condition relative to placebo. There was a time effect for WBSL, LSR and SGA (1 × 1 cm; *F*_(1, 14)_ = 17.438, *p* < 0.001,* η*_p_^2^ = 0.555; *F*_(1, 14)_ = 35.639, *p* < 0.001,* η*_p_^2^ = 0.718; *F*_(1, 14)_ = 4.806, *p* = 0.046,* η*_p_^2^ = 0.256, respectively), but not for SGA (3 × 3 cm; *F*_(1, 14)_ = 4.059, *p* = 0.064,* η*_p_^2^ = 0.225). However, there was no condition × time interaction effect for WBSL (*F*_(1, 14)_ = 1.607, *p* = 0.226,* η*_p_^2^ = 0.103), LSR (*F*_(1, 14)_ = 0.077, *p* = 0.786,* η*_p_^2^ = 0.005), SGA (3 × 3 cm; *F*_(1, 14)_ = 0.035, *p* = 0.854,* η*_p_^2^ = 0.003) and SGA (1 × 1 cm; *F*_(1, 14)_ = 0.182, *p* = 0.676,* η*_p_^2^ = 0.013).

### Partitional calorimetry and skin blood flow

**Fig. 7:**
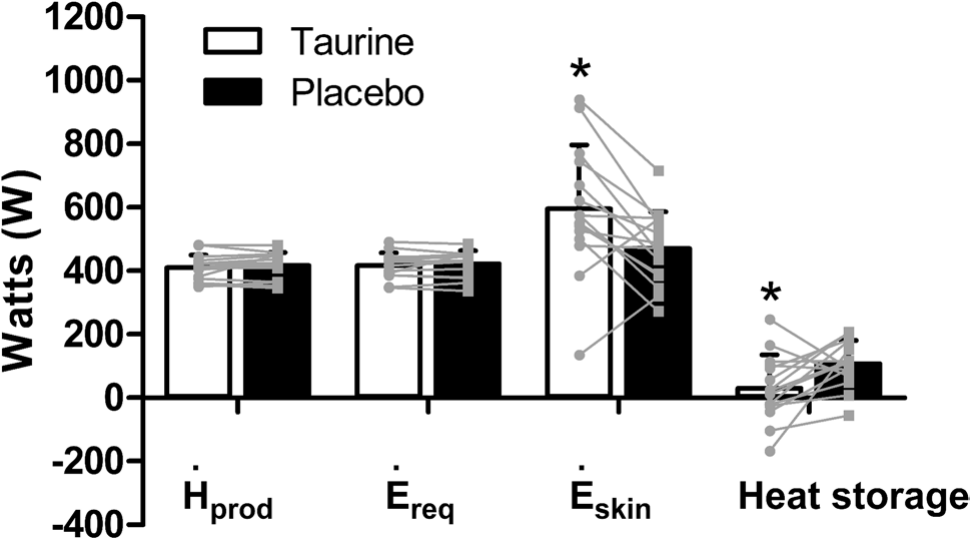
Heat production (*Ḣ*_prod_), evaporative requirement for heat balance (*Ė*_req_), evaporation at the skin surface (*Ė*_skin_) and heat storage during the fixed-humidity exercise period. *Significantly different than placebo (*p* < 0.05)

Evaporation from the skin surface (*Ė*_skin_; *t*_(14)_ = 3.002, *p* = 0.010, Cohen’s* d* = 0.79) was increased and total heat storage decreased (*t*_(14)_ = –2.537, *p* = 0.024, Cohen’s* d* = −0.87) in the taurine condition relative to placebo during the fixed-humidity exercise period (Fig. 7). Calculated SkBF (Taurine = 4.07 ± 1.02; Placebo = 4.31 ± 1.70; *p* = 0.650) did not differ between conditions during the fixed-humidity exercise period.

### Perceptual measurements

Thermal comfort, TS and RPE increased with time in both conditions (*F*_(2.044, 28.614)_ = 18.623, *p* < 0.001,* η*_p_^2^ = 0.571; *F*_(1.901, 26.618)_ = 22.955, *p* < 0.001,* η*_p_^2^ = 0.621; *F*_(1.834, 25.669)_ = 28.562, *p* < 0.001,* η*_p_^2^ = 0.671, respectively); however, there was no main effect for condition (*F*_(1, 14)_ = 0.329, *p* = 0.575,* η*_p_^2^ = 0.023; *F*_(1, 14)_ = 1.072, *p* = 0.318,* η*_p_^2^ = 0.071;* F*_(1, 14)_ = 0.639, *p* = 0.437,* η*_p_^2^ = 0.044, respectively). There was also no interaction effect for TC (*F*_(2.681, 37.536)_ = 1.753, *p* = 0.177,* η*_p_^2^ = 0.111) or TS (*F*_(3.263, 45.686)_ = 1.800, *p* = 0.156,* η*_p_^2^ = 0.114); however, there was for RPE (*F*_(2.445, 34.225)_ = 3.910, *p* = 0.023,* η*_p_^2^ = 0.218). Post-hoc pairwise analysis revealed that RPE was only higher in the taurine condition in the last stage of the ramped-humidity exercise period (Taurine = 11 ± 2; Placebo = 10 ± 2; *p* = 0.035).

### Plasma volume and plasma (tau) concentration

Change in plasma volume across the trial period (Taurine = 0.19 ± 8.44%; Placebo = −1.86 ± 7.67%; *t*_(14)_ = 0.903, *p* = 0.382, Cohen’s* d* = 0.25), did not differ between conditions. Plasma taurine concentration, both corrected and uncorrected for plasma volume, was higher (*F*_(1, 10)_ = 5.266, *p* = 0.045,* η*_p_^2^ = 0.345; *F*_(1, 10)_ = 6.389, *p* = 0.030, *η*_p_^2^ = 0.390, respectively) in the taurine *vs* placebo condition. There was a condition x time interaction effect for corrected and uncorrected plasma taurine concentration (*F*_(1, 10)_ = 20.212, *p* = 0.001,* η*_p_^2^ = 0.669; *F*_(1, 10)_ = 20.918, *p* = 0.001, *η*_p_^2^ = 0.677, respectively). Post-hoc pairwise analysis revealed that both corrected and uncorrected plasma taurine concentration was only higher in the taurine condition post-trial (254 ± 198 µM *vs* 82 ± 59 µM*, p* = 0.011; 257 ± 183 µM *vs* 87 ± 70 µM,* p* = 0.006).

## Discussion

We investigated the effects of 8-day oral taurine supplementation on sweating responses, *T*_core_, calorimetric heat transfer components and delta plasma volume during prolonged low-intensity exercise of a fixed *Ḣ*_prod_ at both fixed and incremental ambient vapour pressure. In acceptance of our hypothesis, taurine supplementation increased parameters of sweating (WBSL, LSR and SGA), which delayed the upward inflection of *T*_core_, denoted by a greater *P*_crit_ in the taurine condition compared to placebo. However, contrary to our hypothesis, there was no effect on plasma volume or *T*_core_ and the augmentation of the sweating response appeared to occur earlier following exercise onset, with a trend for larger changes in WBSL and LSR observed during the fixed-humidity compared to the ramped-humidity exercise period. Despite these latter results, calorimetric modelling during fixed-humidity exercise demonstrated increased latent heat dissipation (*Ė*_skin_) and, consequently, decreased heat storage in the taurine condition compared to placebo, which is of critical importance to the findings of the current study. Thus, oral taurine supplementation appears to acutely elicit thermoregulatory benefits during low-intensity, fixed *Ḣ*_prod_ exercise in the heat, which is related to enhanced evaporative heat exchange, secondary to an accentuated sweating response early in the exercising period.

The increases in WBSL (~ 26.6%) demonstrated during the initial fixed-humidity exercise period are similar to changes reported in WBSL in response to heat acclimation (23%; Poirier et al. 2016), thus suggesting that oral taurine supplementation also stimulates a systemic sweating response. While increases in LSR (~ 15.5%) were considerably lower than demonstrated with heat acclimation (30%; (Ravanelli et al. 2018), it is comparable with the 12.7% increase reported during higher intensity cycling in the heat following an acute taurine dose (Page et al. 2019). Interestingly, a greater recruitment of sweat glands was found in the current study following taurine supplementation compared to placebo [1 × 1 cm (32.2%) and 3 × 3 cm (22.1%)], which is in a similar range to the changes reported following 8 days of heat acclimation involving 90-min treadmill walking at 70% maximum HR in environmental conditions of 38 °C and 65% RH (27.9%; Ravanelli et al. 2018). These changes observed using the absorbent patch and modified iodine-paper techniques are above their established coefficient of variation (12.8 ± 4.8% and 15.9–24.1 ± 9.6–10.8%, respectively) when exercising at 200 W/m^2^ *Ḣ*_prod_ (Peel et al. 2022), demonstrating a genuine increase in the response. It is noteworthy that the augmented sweating responses reported herein during low-intensity exercise occurred prior to any substantial changes in *T*_core_, indicating a marked temporal mismatch between the internal thermal stimulus and the enhanced sudomotor responses. However, it should be noted that changes in *T*_core_ and sweating do not always correspond precisely, due to interplay with additional drivers of the sweating response (Bain et al. 2011; Cramer et al. 2012; Jay et al. 2011). Indeed, there were substantially lower effects of taurine on WBSL (5.1% increase), LSR (7.8% increase) and SGA (1 × 1 cm, 29.9%; 3 × 3 cm, 17.1%) during the ramped-humidity exercise period, compared to earlier in the trial. Therefore, advancing upon previous reports (Page et al. 2019), we demonstrate here that oral taurine supplementation stimulates early onset sweating through increased recruitment of sweat glands during exercise in the heat. This apparent early onset of sweating is somewhat different to strategies that have established effects on thermal sweating, such as heat acclimation, where greater magnitude and more sustained sweating responses are apparent (Ravanelli et al. 2018). Nevertheless, the potential importance of this finding to human thermoregulation is underscored by the translation to an improved evaporative cooling capacity (*Ė*_skin_) in the taurine condition compared to placebo (595 W vs 470 W; 27% difference) and lowered rate of heat storage (30 W vs 108 W; 72% difference).

Owing to the nature of the current experimental protocol, we were also able to determine the effect of taurine supplementation on the breakpoint of compensability during the ramped-humidity exercise period. This threshold is important because, without changes to the exercising intensity or thermal stress, an early transition (such as that found with unacclimated people; Ravanelli et al. 2018) will lead to progressive increases in *T*_core_ until critical temperatures are reached. We revealed that taurine delayed the point of uncompensable heat stress, denoted by a rightward shift in the *P*_crit_ value (Fig. 4; 25.0-mmHg vs 21.7-mmHg). A compensable state was maintained to a higher ambient vapour pressure, most likely due to the earlier greater evaporative cooling. Therefore, the early effects of taurine on evaporative heat transfer in the fixed-humidity trial appear to cause a latent enhancement in heat tolerability, which manifested only in response to a progressive increase in the ambient humidity during the ramped segment. Despite this, *T*_core_ was not significantly different between conditions at the breakpoint of compensability (*P*_crit_) or at any timepoint throughout the trial. There was, however, a trend for a lower core temperature in the taurine condition during both exercise periods, indicating a sustained reduction in heat storage, as demonstrated above.

A novel aspect, and advantage, of the current study was the experimental control of *Ḣ*_prod_ and *Ė*_req_, which are known drivers of thermal sweating (Cramer and Jay 2014, 2016; Gagnon et al. 2013; Peel et al. 2022). This control of the work intensities, metabolic profile and environmental constraints was important, since it is feasible that the 8-day taurine supplementation period could have affected the metabolic response to exercise (Rutherford et al. 2010; Simmonds et al. 2022; Zhang et al. 2004b) and subsequent self-paced work-rates if other exercise models were adopted. Given that the supplementation periods occurred prior to the fixed-humidity and ramped-humidity trials, poor control of these factors would have been sufficient to explain any changes in sweating response. That taurine supplementation did not meaningfully change $\dot{V}$O_2max_ or the $\dot{V}$O_2_-WR relationship in the incremental tests that preceded the heat trials, as well as maintaining its effect on thermal sweating responses under control of *Ḣ*_prod_ and *Ė*_req_, experimentally rules out changes in whole-body metabolism as a viable explanation. However, it remains possible that taurine elicited an endurance training-like effect, as reported previously (Waldron et al. 2018a, 2019; Zhang et al. 2004b) that was not recognised by the limited assessment of these characteristics herein. Indeed, endurance training is known to induce partial heat acclimation (Kobayashi et al. 1980), with lower *T*_core_, increased SGA and sweat rates following 8-weeks of aerobic training (Ravanelli et al. 2018). Therefore, it remains possible that a currently unrecognised enhancement of the endurance phenotype is partly responsible for a change in the sweating response after taurine supplementation.

Given the involvement of numerous physiological mechanisms in stimulating eccrine sweat production (Shibasaki and Crandall 2010) and the wide-spread bioavailability of taurine (HuxTable 1992), there are some potential mechanisms that require further investigation. A primary biological role of taurine is as an osmolyte (Cuisinier et al. 2002; HuxTable 1992), with taurine transporters (TauT) ubiquitously expressed in many tissues, including the kidney (Baliou et al. 2020; Han et al. 2000, 2006; Ito et al. 2010). Thus, taurine has potential to affect fluid regulation at the cellular and organ level. Indeed, exercise-induced changes in endogenous plasma taurine concentrations are related to osmoregulatory function during endurance exercise (Cuisinier et al. 2001; Ward et al. 1999), where it is actively extruded from skeletal muscle cells (Graham et al. 1991, 1995) to maintain intracellular osmolality (Lang et al. 1998; Sejersted and Sjøgaard 2000; Stutzin et al. 1999). However, endogenous taurine (i.e. without oral supplementation) has not been reported to affect plasma volume (Cuisinier et al. 2002) and, herein, we also found no difference in the plasma volume changes across the exercise period. However, given that the taurine condition lost a greater amount of fluid through sweating, and fluid ingestion was equal between conditions, the matching of plasma volume changes between conditions perhaps indicates a regulatory role of exogenous taurine in maintaining plasma volume, despite the additional fluid losses. Given that taurine is extruded from myocytes to the extracellular space during exercise to prevent cell swelling (Stutzin et al. 1999), a greater osmotic gradient and fluid availability in the extracellular compartments to maintain plasma volume is entirely feasible. The consequence of these findings on all fluid compartments during exercise in the heat is uncertain, however, and requires further research. It is unfortunate that plasma volume measurements were not more frequent, as earlier transient changes might have occurred in tandem with the early sweating onset but will not have been identifiable. Theoretically, plasma volume maintenance may have augmented sweating via preservation of SkBF (Nagashima et al. 1998; Nielsen et al. 1984), fluid availability and supply to the sweat gland (Fortney et al. 1981; Wong and Hollowed 2017) or a change in osmoreceptor or baroreceptor signalling (Mack et al. 1995; Shibasaki et al. 2003).

Estimated whole-body SkBF was not different between conditions. This result was unanticipated, as taurine acts peripherally, as a vaso-relaxant, with TauT abundantly expressed in vascular smooth muscle (Liao et al. 2007). Supplementation has been demonstrated to improve both endothelium dependant and independent vasodilation (Maia et al. 2014; Sun et al. 2016; Ulusoy et al. 2017), through increased nitric oxide bioavailability, restoration of vascular redox homeostasis and calcium activated potassium channel opening action (Maia et al. 2014; Ulusoy et al. 2017). Its apparent homeostatic function appears to play a role in vascular tone, by promoting both vasodilation and vasoconstriction, to increase blood flow during ischemia, hypoxia or heat stress and maintain blood pressure (Nishida and Satoh 2009). An association between sweating and SkBF has been established (Van Beaumont and Bullard 1965; Nadel et al. 1971a, 1971b; Brengelmann et al. 1973), suggesting a functional inter-relationship (Wong and Hollowed 2017). However, findings by Ravanelli et al. (2017) demonstrate that increases in SkBF are not a prerequisite for increases in LSR, at least acutely. This is supported by the results herein, as the similarity in estimated whole-body SkBF between the taurine and placebo condition demonstrates no apparent vascular effect, despite the concomitant increased sweating response. While this may be the case, attenuation of SkBF through arterial occlusion (MacIntyre et al. 1968; Collins et al. 1959) or pharmacological blockade (Wingo et al. 2010) reduces the sweating response during heat stress, suggesting a requirement of SkBF for sustained sweating. Future analysis of cutaneous vascular conductance using laser Doppler flowmetry would more conclusively establish whether additional SkBF is required to continue to supply the sweat gland during prolonged periods of sweating and whether taurine supplementation facilitates this.

This study characterised multiple measures of the sweating response (WBSL, LSR and SGA), which demonstrated changes of a large magnitude after taurine supplementation. This enhancement occurring in the earlier segments of the current trial, prior to any marked heat strain, requires mechanistic reasoning and may be explained by a centrally mediated alteration in thermoregulatory set-point. Indeed, whilst taurine supplementation did not significantly affect *T*_core_, there were clear effects on the domain of compensability in the current study, and the enhanced early sweating response occurred alongside increased sweat gland recruitment, providing further evidence for an alteration in the thermoregulatory feedback loop. This has been suggested previously in exercising humans to explain early onset of sweating in response to high-dose (50 mg/kg/bm) taurine supplementation (Page et al. 2019). Additionally, another potential mechanism for the increased sweating observed is through taurine antagonism of antidiuretic hormone (ADH) or arginine vasopressin (AVP). Vasopressin is an antidiuretic hormone produced in the supraoptic nucleus of the hypothalamus and released by the posterior pituitary gland in response to plasma hyperosmolality (Cunningham and Sawchenko 1991; Bourque et al. 1994; Richard and Bourque 1995). Both potential mechanisms can be linked to taurine’s role as a neuromodulator, where it acts as a glycine and GABA receptor agonist and appears to have multiple roles in maintaining homeostasis during periods of perturbation (Jia et al. 2008; Schmieden et al. 1992; Hussy et al. 1997). Indeed, it functions to protect neurons from toxicity by modulating thalamic network activity under conditions of homeostatic derangement, which are associated with severe pathological conditions (Jia et al. 2008) and may be extended to transient states of heat stress. It has been identified in the animal model that GABA and taurine are released from some hypothalamic cells into the cerebrospinal fluid during thermal strain, which coincides with reductions in *T*_core_ (Frosini et al. 2000). Following oral supplementation, the increased plasma taurine is available to cross the blood–brain barrier via TauT (Kang 2002), and act on hypothalamic regions of the brain, potentially interacting with a specific taurine binding site (i.e. putative Taurinergic pathway) or GABA receptors (Frosini et al. 2003; Quéva et al. 2003). Whilst it remains speculative, in the exercising, thermally-stressed human, we propose that the established cryogenic effect of these pathways (Frosini et al. 2003; Elhussiny et al. 2021) may translate to enhanced sudomotor function, as the major effector response to heat stress. Similarly, taurine’s release from the hypothalamus in response to plasma hypoosmolality and its agonism of glycine receptors is suggested to exert an inhibitory effect on ADH secretion (Hussy et al. 1997; Miyata et al. 1997; Deleuze et al. 1998), thereby promoting increased fluid loss. Due to their many similarities, it has been suggested that ADH may facilitate fluid reabsorption at the sweat gland, as it does in the kidney, through its action on vasopressin subtype 2 receptors (V2 receptors), promoting fluid and sodium reabsorption to maintain fluid homeostasis (Agu 2017; Hew-Butler 2010; Baker 2019). As such, exogenous taurine supplementation may supress the release of ADH and attenuate water reabsorption at the sweat gland, leading to greater fluid loss. In the rat model, subcutaneous injection of ADH reduced initial sweat rate by 50%, suggesting a role for ADH in regulating the sweating response (Quatrale and Speir 1970). Further, in exercising humans, a positive association between plasma ADH and sweat sodium concentrations has been reported, indicating it may have a potential role in fluid retention at the sweat gland (Hew-Butler 2010). Nevertheless, several studies both augmenting and suppressing ADH have observed no significant change in sweat rate during exercise or heat exposure (Pearcy et al. 1956; Senay and Van Beaumont 1969; Ratner and Dobson 1964; Gibiński et al. 1979; Taussig and Braunstein 1973; Allen and Roddie 1974; Hew-Butler et al. 2014). However, none of these studies have investigated the effect of ADH on the sweating response during exercise in the heat when fluid intake is regulated, and other drivers of the sweating response are controlled (e.g. *Ḣ*_prod_ and *Ė*_req_). Therefore, this remains a plausible pathway in which exogenous taurine supplementation augments the thermal sweating response and requires further investigation. Whilst the above mechanisms of action provide a potential explanation for the current results, it is reasonable to suggest that the increased sweating response occurs owing to a combination of several factors, which is consistent with the numerous biological roles ascribed to taurine (HuxTable 1992). In-vivo investigation of the above central mechanisms is likely to be challenging but could be addressed in future research.

High environmental heat stress alongside physical exertion where compensatory limits for heat dissipation are exceeded, pose a risk for serious heat illness due to inexorable increases in *T*_core_ (Howe and Boden 2007; Epstein and Yanovich 2019). Athletes and military personnel are often required to perform endurance exercise in such conditions (Ely et al. 2008; Racinais et al. 2015; World and Booth 2008; Parsons et al. 2019) and, therefore, a strategy (e.g. taurine supplementation) to help offset this rise in *T*_core_, and the risk of heat illness could be important in reducing its prevalence. To the authors’ knowledge, there are no known serious side effects of taurine supplementation at doses of up to 10-g (Shao and Hathcock 2008). Therefore, supplementing 50 mg/kg/bm of taurine prior to heat exposure might be a useful strategy to offset the deleterious effects of heat stress among healthy populations. However, further research is required to better understand the efficacy of its thermoregulatory role in applied settings. Thus, findings that oral taurine supplementation can acutely increase the sweating response, augment evaporative heat transfer, and reduce heat storage during low-intensity exercise in the heat has many promising future applications.

## Conclusion

Eight days of oral taurine supplementation (50 mg/kg/bm) increased sweating responses (WBSL, LSR and SGA) during low-intensity exercise of a fixed *Ḣ*_prod_ in the heat. The subsequent enhanced evaporative cooling (*Ė*_skin_) and reduced heat storage delayed the upward inflection in *T*_core_—represented by a greater *P*_crit_—demonstrated during the exercise period of incremental ambient vapour pressure. Despite this, there was only a trend towards a lower *T*_core_ throughout the trial, and reduced changes in WBSL and LSR during the ramped-humidity exercise period. This apparent early augmentation of the sweating response appears to offer thermoregulatory benefits for latter parts of an exercising period. These findings have potential implications for both athletes and military personnel performing exercise in hot environmental conditions that permit sufficient latent heat transfer. The experimental control of the thermal drivers of sweating (*Ḣ*_prod_ and *Ė*_req_) suggests that other mechanisms are likely to be responsible for the observed increase in sweating. We suggest several possibilities to direct future investigations, which will help to elucidate the mechanistic actions of taurine during exercising-heat stress.

### Supplementary Information

Below is the link to the electronic supplementary material.

Supplementary file1 (PDF 139 KB)

## Abbreviations

ADH: Antidiuretic hormone
AVP: Arginine vasopressin
BSA: Body surface area
*Ė*_max_: Maximal evaporative heat transfer capacity
*Ė*_req_: Evaporation required to maintain heat balance
*Ė*_skin_: Evaporative heat transfer
*Ḣ*_prod_: Heat production
HR: Heart rate
LSR: Local sweat rate
*Ṁ*: Metabolic energy expenditure
*P*_crit_: Breakpoint of uncompensability
RER: Respiratory exchange ratio
RH: Relative humidity
RM-ANOVA: Repeated measures analysis of variance
RPE: Rating of perceived exertion
SGA: Sweat gland activation
TauT: Taurine transporters
TC: Thermal comfort
*T*_core_: Core temperature
*T*_db_: Dry bulb temperature
TS: Thermal sensation
*T*_sk_: Skin temperature
$\dot{V}$O_2max_: Maximal oxygen uptake
V2 receptors: Vasopressin subtype 2 receptors
WBSL: Whole-body sweat loss
Wk: Mechanical work

## Declarations

### Data availability

The datasets generated during and/or analysed during the current study are available from the corresponding author on reasonable request.
