# Supplementary material for: The effect of 8-day oral taurine supplementation on thermoregulation during low-intensity exercise at fixed heat production in hot conditions of incremental humidity
Source: Eur J Appl Physiol. 2024 Apr 6;124(9):2561–76. doi: 10.1007/s00421-024-05478-3 (PMC11365861; doi:10.1007/s00421-024-05478-3)
Supplement: Supplementary file 1 — Supplementary file1 (PDF 139 KB) [file 421_2024_5478_MOESM1_ESM.pdf]

Title: The effect of eight-days oral taurine supplementation on thermoregulation during low-intensity exercise at fixed heat production in hot conditions of incremental humidity

European Journal of Applied Physiology

Authors: Jennifer S. Peel[1]*, Melitta A. McNarry[1], Shane M. Heffernan[1], Venturino R. Nevola[1,2], Liam P. Kilduff[1,3], Kathryn Coates[4], Ed Dudley[4], Mark Waldron[1,3,5]

[1]A-STEM Centre, Faculty of Science and Engineering, Swansea University, Swansea, UK.

[2]Defence Science and Technology Laboratory (Dstl), Fareham, Hampshire, UK.

[3]Welsh Institute of Performance Science, Swansea University, Swansea, UK.

[4]Swansea University Medical School, Faculty of Medicine, Health and Life Science, Swansea University, Swansea, UK.

[5]School of Health and Behavioural Sciences, University of the Sunshine Coast, Queensland, Australia.

* = corresponding author

Email: 856558@swansea.ac.uk

ORCID: 0000-0002-7651-8979

Supporting information

Equations list

$$H_{dry\ skin} = C_{skin} + R_{skin} + K_{skin}\ [W] \qquad \text{(equation 12)}$$

$H_{dry\ skin,}$ dry heat exchange at the skin surface; C_{skin}, convection; R_{skin}, radiation; K_{skin}, conduction

$$C_{skin} + R_{skin} = \frac{(t_{sk} - t_0)}{\left(R_{cl}\frac{1}{h \times fcl}\right)} \times A_D\ [W] \qquad \text{(equation 13)}$$

t_{sk}, skin temperature; t_o, operative temperature; R_{cl}, dry heat transfer of clothing; h, combined convective heat transfer coefficient; f_{cl}, clothing area factor; A_D, body surface area

$$t_0 = \frac{h_r t_r + h_c t_a}{h_r + h_c}\ [W] \qquad \text{(equation 14)}$$

h_r, radiative heat transfer coefficient; t_r, radiant temperature; h_c, convective heat transfer coefficient; t_a, ambient air temperature

$$h = h_c + h_r \ [W/m^2/K]$$ (equation 15)

$$h_c = 8.3 \times v_{air}^{0.6} \ [W/m^2/K]$$ (equation 16)

V_{air}, ambient air velocity

$$h_r = 4\, \varepsilon\, \sigma \frac{A_r}{A_D}\left(273.2 + \frac{t_{sk}+t_r}{2}\right)^3 \ [W/m^2/K]$$ (equation 17)

ε, non-dimensional emissivity of the body surface; σ, Stefan-Boltzmann constant; A_r/A_D, fraction of the body surface participating in radiative heat transfer

$$H_{res} = C_{res} + E_{res} \ [W]$$ (equation 18)

H_{res}, respiratory heat loss; C_{res}, convective respiratory heat loss; E_{res}, evaporative respiratory heat loss

$$C_{res} = 0.001516 \times M(28.56 + 0.641 \times Pa - 0.885 \times t_a) \ [W]$$ (equation 19)

M, metabolic energy expenditure; P_a, vapor pressure of inspired air

$$E_{res} = 0.00127 \times M(59.34 + 0.53 \times t_a - 11.63 \times Pa \ [W]$$ (equation 20)

$$Pa = \frac{6.116441 \times 10^{\left(\frac{7.5911386 \times t_a}{t_a + 240.7263}\right)} \times \frac{\%RH}{100}}{10} \ [kPa]$$ (equation 21)

%RH, relative humidity

$$\dot{E}_{max} = \frac{(P_{skin,sat} - P_a)}{R_{e,cl} + \frac{1}{h_e \times f_{cl}}} \times A_D \ [W]$$ (equation 22)

$R_{e,cl}$, evaporative resistance of clothing;

$$P_{skin,sat} = \frac{\frac{(EXP(18.956 - (4030.18)}{t_{sk} + 235}}{10} \ [kPa]$$ (equation 23)

$$h_e = (16.5 \times h_c) \ [W]$$ (equation 24)

$$H_{evap\_skin} = WBSR \times \lambda \times \frac{S_{eff}}{60} \ [W]$$ (equation 25)

WBSR (whole-body sweat rate), based on body mass changes over time (g/min); λ is the latent heat vaporisation of sweat (2426 J/g)

$$S_{eff} = 1 - \frac{{\omega_{req}}^2}{2} \ [ND]$$ (equation 26)

$$\omega_{req} = \frac{\dot{E}_{req}}{\dot{E}_{max}} \ [ND]$$ (equation 27)

See Cramer and Jay (2019) for further information regarding partitional calorimetry, these equations and their components.

Cramer MN, Jay O (2019) Partitional calorimetry. Journal of Applied Physiology 126(2):267-77.
